# Supplementary figures and images for: Dose-Dependent Effect of DNA Vaccine pVAX-H5 Encoding a Modified Hemagglutinin of Influenza A (H5N8) and Its Cross-Reactivity Against A (H5N1) Influenza Viruses of Clade 2.3.4.4b
Source: Viruses. 2025 Feb 27;17(3):330. doi: 10.3390/v17030330 (PMC11946855; doi:10.3390/v17030330)

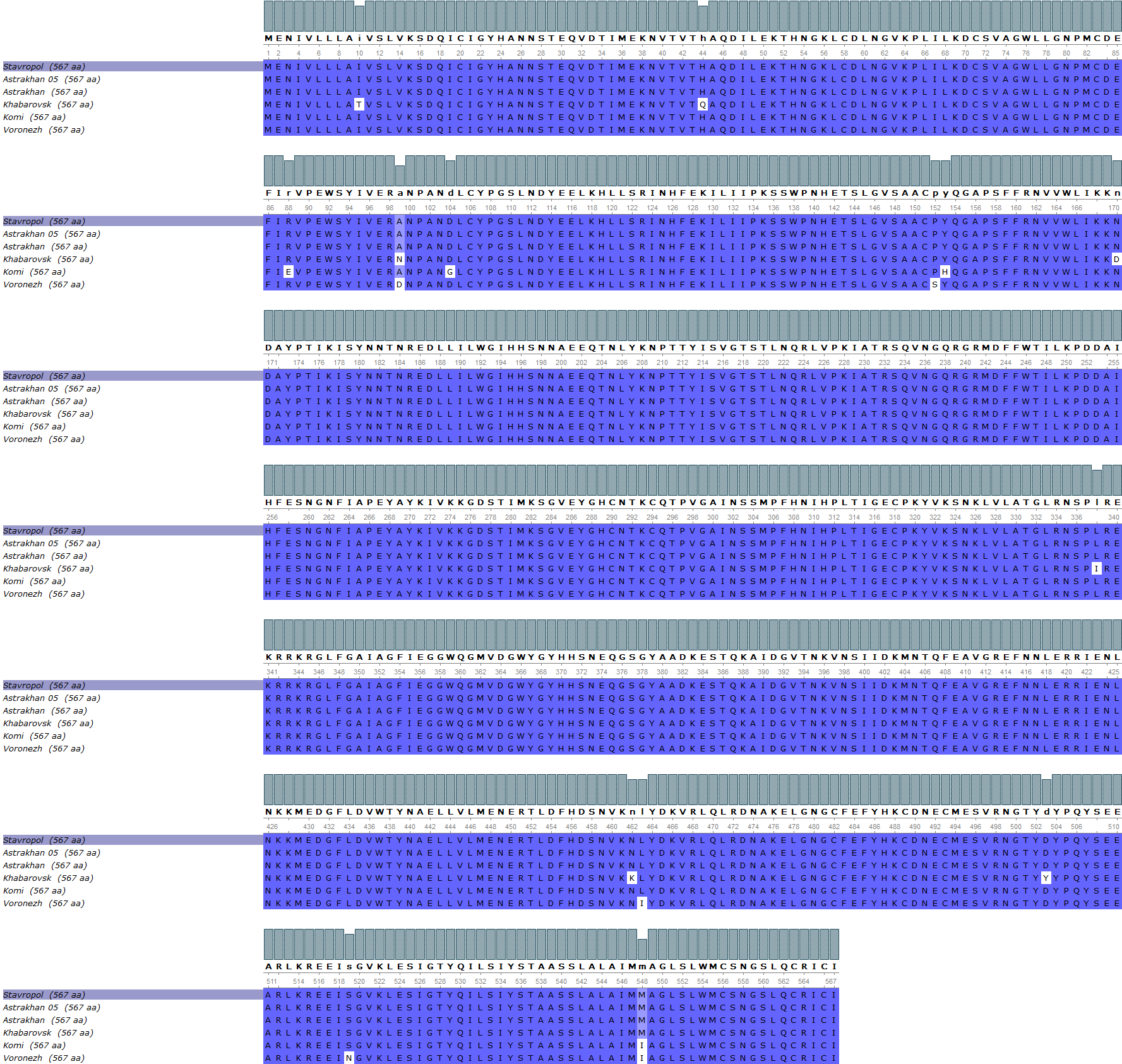

Supplement: Supplementary file 1 [file viruses-17-00330-s001.zip › viruses-3376804-supplementary.png]
